# Taking a spiritual history in the oral oncology setting

**DOI:** 10.1007/s00520-026-11215-7

**Published:** 2026-09-18

**Authors:** Alan Roger Santos-Silva, Marco Magalhaes, Camila Barcellos Calderipe, Marcio Ajudarte Lopes, Eric V. Ávila-Pires, Joel B. Epstein

**Affiliations:** 1 Department of Oral Diagnosis, Piracicaba Dental School, University of Campinas (UNICAMP), Av. Limeira, 901, Bairro Areão, Piracicaba, São Paulo Brazil; 2 Oral and Maxillofacial Pathology and Oral Medicine, Faculty of Dentistry, University of Toronto, Toronto, ON Canada; 3 NUPES, Research Center in Spirituality and Health, School of Medicine, Federal University of Juiz de Fora (UFJF), Juiz de Fora, Minas Gerais Brazil; 4 City of Hope Comprehensive Cancer Center, Duarte, CA USA; 5 Cedars Sinai Health System, Los Angeles, CA USA

**Keywords:** Spiritual history, Spirituality, Oral cancer

## Abstract

**Background:**

Spirituality is recognized as a dimension of health and is increasingly incorporated into supportive cancer care settings. Patients with oral cancer frequently experience profound functional, psychosocial, and existential challenges associated with their diagnosis and treatment, often leading to unmet spiritual needs. Taking a spiritual history provides an approach to identify beliefs, values, and coping resources that influence quality of life and clinical decision-making.

**Objective:**

To synthesize evidence-based strategies for assessing patients’ spirituality within oral oncology practice, emphasizing spiritual history tools, clinical impact, and educational needs.

**Methods:**

A narrative review was conducted using the SANRA tool as guidelines, focusing on peer-reviewed literature, clinical frameworks, and educational interventions addressing spiritual assessment in patients with serious illness, those relevant to multiprofessional cancer care.

**Results:**

Instruments such as FICA and HOPE demonstrated positive effects on trust, communication, and adherence to treatment. Despite these benefits, the spirituality assessment remains limited in oral oncology. Educational models have shown measurable improvements in clinician confidence and skill acquisition.

**Conclusion:**

These tools make it feasible for professionals in oral oncology to take patients’ spiritual history, which enhances holistic care, strengthens multidisciplinary alignment, and supports patient-centered outcomes. Implementation requires initiatives and standardized referral to address identified spiritual needs.

## Introduction

The World Health Organization recognizes spirituality as a dimension of health and acknowledges its integral role in holistic, patient-centered care [1, 2]. It can be defined as the relationship with a transcendent realm considered sacred, the ultimate truth, or reality [3]. However, in clinical practice, it is approached as the aspect of humanity that refers to the way individuals seek and express meaning and purpose and the way they experience their connectedness to the moment, to self, to others, to nature, and to the significant or sacred [4].

Within oral oncology, spirituality is relevant because of the profound and often rapid impact oral cancer has on patients’ identity, communication, social interactions, and daily functioning. Tumor biology, surgical resections, radiation-induced toxicities, chemotherapy, targeted therapy and immunotherapy toxicities, and long-term rehabilitation may result in facial disfigurement, speech impairment, dysphagia, chronic pain, taste loss, and salivary dysfunction, challenges disrupting body image, autonomy, and quality of life [5–7]. In turn, these oral complications impact oral and dental health potentially leading to significant adverse effects. Consequently, patients frequently experience emotional distress, social withdrawal, fear of recurrence, financial distress, and existential uncertainty, which may surface as tears, silence, expressions of hopelessness, or resistance to treatment [8–13].

In this context, taking a spiritual history offers a pathway to explore how individuals attribute meaning to suffering, preserve dignity, and identify sources of resilience during cancer treatment. For many, spirituality functions as a coping mechanism, shaping pain tolerance, motivation to complete therapy, adherence to follow-up, and willingness to engage in complex rehabilitation processes [14–17]. Despite its clinical importance, including for the health system, spiritual assessment remains underutilized during multiprofessional care [18–23].

Therefore, this narrative review aimed to synthesize evidence-based strategies for assessing spirituality within oral oncology practice, emphasizing spiritual history tools, clinical impact, and educational needs.

## Methods

This brief narrative review used the SANRA criteria [24]. Relevant peer-reviewed studies, guidelines, and book chapters were selected based on their contribution to evidence-based tools, clinical outcomes, and educational interventions related to spiritual history-taking. The search terms used were “oral cancer,” “spirituality,” “religiosity,” “spiritual care,” “spiritual well-being,” “pastoral care,” “religious support,” “faith-based care,” “spiritual counselling,” and “holistic care.”

## Results

### Spiritual history tools and structured approaches

Evidence-based frameworks have been developed to support structured spiritual history-taking, including FICA, HOPE, SPIRIT, FAITH, the tool endorsed by the Royal College of Psychiatrists, and the Patient-Centered Clinical Method [14, 15, 25–29] (Table 1). These instruments assess patients’ beliefs, religious or spiritual practices, sources of strength, and how these influence coping and medical decision-making [15, 30]. Among these, FICA and HOPE are the most widely used, validated, and adaptable across diverse healthcare environments, with studies confirming their reliability and feasibility in routine practice—for example, FICA takes only about 2 min (Tables 2 and 3) [14, 15, 25, 31, 32]. These tools provide structured pathways for clinicians to explore spirituality respectfully and efficiently within boundaries.

**Table 1:** Frameworks for structured spiritual history-taking

Tool	Acronym (expanded)	Domains assessed	Distinctive characteristics
FICA^14^	F—Faith I—Importance C—Community A—Address in care	Beliefs and meaning; perceived importance; community and support; integration into care	Brief and easy to administer in routine encounters; commonly referenced in clinical literature; has published studies examining its clinical applicability
HOPE^25^	H—Hope (sources of hope/strength) O—Organized religion P—Personal spirituality/practices E—Effects on medical care	Sources of hope and resilience; religious affiliation; personal practices; influence on care and decisions	Flexible structure; begins with broader existential questions; frequently cited in palliative and multicultural care contexts
SPIRIT^26^	S—Spiritual belief system P—Personal spirituality I—Integration with community R—Rituals/restrictions I—Implications for care T—Terminal events planning	Belief system; personal spirituality; community connections; ritual practices; implications for care; end-of-life considerations	More detailed and comprehensive than most mnemonic tools; includes items on medical restrictions and terminal planning
FAITH^27^	F—Faith/beliefs A—Availability/accessibility of spiritual resources I – Importance T—Treatment implications H—Heart (core meaning)	Beliefs; accessibility of spiritual resources; personal importance; implications for treatment; core meaning	Concise tool with limited published research on its use; emphasizes integration of spiritual concerns into treatment planning; less widely used compared to FICA, HOPE and SPIRIT
Royal College of Psychiatrists assessment^28^	No acronym	Spiritual history; significant experiences; practices and rituals; spiritual resources; spiritual needs; influence on mental health and treatment	Multidimensional framework primarily designed for psychiatric settings; encompasses spiritual experiences, potential spiritual distress, and sources of support
Patient-Centered Clinical Method^29^	No acronym	Spirituality integrated across the illness experience, biopsychosocial context of the whole person, shared therapeutic planning, and the patient-clinician relationship	Framework-based rather than question-based approach; integrates spiritual exploration throughout the clinical encounter using patient-centered communication; emphasizes patients’ narratives and re-signification of spiritual coping strategies; originally developed for primary care settings

**Table 2:** The FICA spiritual history tool

Letter	Domain	Sample questions
F	**F**aith, belief, meaning	Do you consider yourself spiritual or religious? Do you have spiritual beliefs or practices that help you cope with stress? If no: What gives your life meaning?
I	**I**mportance and **I**nfluence	What importance does your faith or belief have in your life? Do you have beliefs that might influence your health care decisions? If so, are you willing to share those with your health care team?
C	**C**ommunity	Are you part of a spiritual or religious community? Is this of support to you, and how?
A	**A**ddress/**A**ction in care	How should I address these issues in your health care? (Prompts the clinician to develop a plan for spiritual distress or needs.)

Adapted from Puchalski CM. The FICA spiritual history tool #274. J Palliat Med. 2014;17:105–106

**Table 3:** The HOPE questions for spiritual assessment

Letter	Domain	Sample questions
H	Sources of **H**ope, meaning, comfort, strength, peace, love, and connection	What gives you hope, strength, comfort, and peace? What do you hold on to during difficult times? What sustains you and keeps you going?
O	Role of **O**rganized religion	Do you consider yourself part of an organized religion? How important is this to you? What aspects of your religion are helpful and not so helpful to you?
P	**P**ersonal spirituality and **P**ractices	Do you have personal spiritual beliefs independent of organized religion? What aspects of your spirituality or practices do you find most helpful (e.g., prayer, meditation, reading scripture)?
E	**E**ffects on medical care and **E**nd-of-life issues	Has being sick affected your ability to do things that usually help you spiritually? (or affected your beliefs?) As a health care professional, is there anything I can do to help you access the resources that usually help you? Are you worried about conflicts between your beliefs and your medical care? Would it help to speak with a chaplain or spiritual leader? Are you worried about any conflicts between your beliefs and your medical care, or are there any specific practices or restrictions I should know about?

Adapted from Anandarajah G, Hight E. Spirituality and medical practice: using the HOPE questions as a practical tool for spiritual assessment. Am Fam Physician. 2001;63:81–89

### Impact on clinical care and patient outcomes

Evidence shows that most patients view spiritual history-taking positively, particularly when coping with chronic or severe illness. In a systematic review, the majority of samples considered it appropriate for healthcare professionals to ask about spiritual needs in at least some circumstances [33]. In a cross-sectional study, approval rates ranged from 94.0% to 99.8% for the spiritual history questions [34]. The integration of spiritual inquiry into clinical encounters has also been associated with improved provider-patient relationships, increased trust, and greater satisfaction with care [17, 33–36]. In an intervention study, after 3 weeks, patients who received the spiritual intervention showed a greater reduction in depressive symptoms (*F* = 7.57; *p* < 0.01) and greater improvement in quality of life (*F* = 4.04; *p* < 0.05) compared with the control group. In addition, patients who received the intervention reported a more positive perception of the interpersonal care provided by the physician (*F* = 4.79; *p* < 0.05) [37]. In a cluster-randomized trial, patients who reported that spirituality played an important role in their lives showed a 6.2-point difference in mental well-being, as assessed by the SF-12 mental health subscale, compared with the control group (*d* = 0.65; *p* = 0.002). Although the primary outcome, health-related self-efficacy, showed no significant effect (*d* = 0.14; *p* = 0.25), these findings suggest possible benefits of the intervention on mental well-being [38]. In another randomized controlled trial, patients who underwent spiritual assessment showed greater attendance at clinical appointments during the 3-month follow-up period compared with the control group (*t* = − 2.45; *p* = 0.02). Only 8% of patients in the intervention group missed appointments, compared with 26% in the control group (*χ*^*2*^ = 4.97; df = 1; *p* = 0.03) [39]. Notably, brief conversations acknowledging spiritual concerns can foster emotional support and signal clinician openness, without causing distress or discomfort [36, 40].

For oral oncology, beyond diagnostic and treatment phases, patients often experience chronic oral mucosal and dental complications, osteonecrosis, and life-threatening infections. Spiritual concerns may significantly influence coping, pain perception, and treatment acceptance [7, 8, 16, 41–45]. Incorporating tools in these settings may therefore strengthen supportive care and improve patient experience. Spiritual history-taking has the potential to:

- Improve rapport and communication
- Increase trust and satisfaction with care
- Improve mental well-being
- Increase follow-up attendance
- Enhance coping during decision-making

### Barriers and educational strategies

Despite positive outcomes and professional recommendations, spirituality remains insufficiently explored in clinical practices. Common barriers include insufficient time, lack of knowledge and training, discomfort discussing spiritual topics, and uncertainty regarding professional boundaries [18–23, 33]. These challenges may arise early in the management of patients with oral cancer, as some professionals, such as those in dentistry, often lack specific training in psychosocial care. Unfortunately, this may also be the case with other members of the healthcare team.

Educational interventions have demonstrated success in overcoming these limitations. Role-play exercises, standardized patient interactions, and curriculum modules increase healthcare providers’ confidence, communication skills, and feasibility of spiritual assessment [18–21, 23]. Spiritual care competencies can be taught in undergraduate and postgraduate programs, including continuing education environments, with demonstrated improvement in knowledge retention and behavioral application [18, 19, 21–23].

Expanding these initiatives into health training curricula may equip clinicians to more confidently and ethically address spiritual concerns within their scope of care.

### Relevance to oral oncology multiprofessional teams

Dentists and oral medicine specialists are often positioned at the beginning of the care pathway and continue to provide care throughout and after the oncologic treatment. They collaborate with physicians, nurses, nutritionists, and psycho-oncology teams, who may encounter spiritual concerns and emotional distress during patient care. Spiritual history-taking can facilitate appropriate referrals to chaplaincy or mental health services, support shared decision-making, and align treatment with patient values. As supportive care in cancer evolves toward multidisciplinary, patient-centered models, healthcare professionals should be prepared to identify and respectfully address spiritual needs that may affect adherence, coping, and quality of life.

## Conclusions

There is evidence supporting the use of structured frameworks to obtain a spiritual history in clinical practice. These strategies are well-accepted by patients, improve communication and satisfaction, and enhance clinician preparedness to deliver holistic care. Incorporating spiritual assessment into multiprofessional practice, beginning with oral medicine and continuing throughout care, may strengthen supportive oncology care, promote cultural sensitivity, and address a frequently unmet dimension of patient well-being. Recommended actions include the adoption of spiritual history-taking tools and the expansion of targeted education to enhance clinician skills and confidence.

## Data Availability

No datasets were generated or analysed during the current study.
